# Chitin, Chitosan, and Nanochitin: Extraction, Synthesis, and Applications

**DOI:** 10.3390/polym14193989

**Published:** 2022-09-23

**Authors:** Michael Kozma, Bishnu Acharya, Rabin Bissessur

**Affiliations:** 1 Department of Chemistry, University of Prince Edward Island, 550 University Avenue, Charlottetown, PE C1A 4P3, Canada; 2 Department of Chemical and Biological Engineering, University of Saskatchewan, 57 Campus Drive, Saskatoon, SK S7N 5A9, Canada

**Keywords:** crustacean shell, chitin, chitosan, nanochitin, extraction methods, circular bioeconomy

## Abstract

Crustacean shells are a sustainable source of chitin. Extracting chitin from crustacean shells is ongoing research, much of which is devoted to devising a sustainable process that yields high-quality chitin with minimal waste. Chemical and biological methods have been used extensively for this purpose; more recently, methods based on ionic liquids and deep eutectic solvents have been explored. Extracted chitin can be converted into chitosan or nanochitin. Once chitin is obtained and modified into the desired form, it can be used in a wide array of applications, including as a filler material, in adsorbents, and as a component in biomaterials, among others. Describing the extraction of chitin, synthesis of chitosan and nanochitin, and applications of these materials is the aim of this review. The first section of this review summarizes and compares common chitin extraction methods, highlighting the benefits and shortcomings of each, followed by descriptions of methods to convert chitin into chitosan and nanochitin. The second section of this review discusses some of the wide range of applications of chitin and its derivatives.

## 1. Introduction

Chitin is the second-most abundant natural polysaccharide in the world, exceeded in mass only by cellulose. Chitin is a polymer composed of β-1,4-linked *N*-acetyl-D-glucosamine. Three polymorphic forms of chitin exist, based on the arrangement of polymer chains: α-chitin refers to when the chains lie antiparallel to one another, β-chitin refers to chitin with parallel chains, and γ-chitin is a combination of α- and β-chitin, with some chains parallel and some chains antiparallel to one another [1] (pp. 90–93). Chitin can be found in the cell walls of fungi, the exoskeletons of arthropods, and in some mollusks; α-chitin is the most abundant in nature and is commonly sourced from crustacean shells [1,2]. The high degree of crystallinity and strong hydrogen bonds between chitin chains render chitin insoluble in water and many organic solvents; despite this, chitin has found applications in many fields, including textiles, paper making, medicine, and wastewater treatment [1,2,3,4].

**Figure 1:**
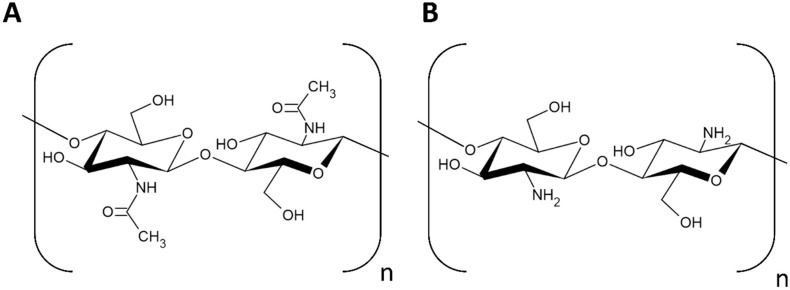
Structure of: (**A**) chitin and (**B**) chitosan.

Chitosan is the partially deacetylated form of chitin, where a fraction of the N-acetyl-D-glucosamine has been converted into D-glucosamine. The ratio of D-glucosamine to N-acetyl-D-glucosamine in chitosan is referred to as the degree of deacetylation (DD). Fully acetylated and fully deacetylated chitin do not exist in nature. The necessary DD to be considered chitosan varies, but a DD of 50% or greater is generally considered chitosan. Chitosan is the only natural cationic polymer [3,5]. Chitosan is considered the most valuable derivative of chitin due to its myriad applications in a wide range of fields, including medicine, agriculture, and its role in forming biodegradable plastics. Chitosan is broadly appreciated for its biocompatibility, biodegradability, antimicrobial properties, and low toxicity in the environment [2,5,6]. Chitin and chitosan are pictured in Figure 1.

Approximately 9.4 million tonnes of crustaceans were fished through aquaculture in 2018. Wild fishing was an additional 6.3 million tonnes [7,8]. An estimated 6 to 8 million tonnes of crustacean shell waste are discarded annually [9]. The majority of chitin sourced from crustacean shells is extracted from shrimp and crab shells, though the potential of other species is an active field of research. Independent of origin species, waste crustacean shells are considered a sustainable source of biomass for the extraction of chitin, and therefore production of chitosan [2,3,5,10,11].

This review summarizes developments in the extraction of chitin from crustacean shells, production of chitosan and nanochitin, and applications for each of these materials. prior reviews have delved extensively into extraction methods for chitin and related materials; some describe a single suite of methodologies, whether they be chemical or biological, and some approach the field as a whole [5,10,11,12,13,14,15]. Other reviews have discussed work related to applications of these materials, including broad reviews providing surveys on a wide range of applications and more focused reviews describing specific fields, such as drug delivery, tissue engineering, and energetic applications [3,6,14,15,16,17,18,19,20,21]. The objective of this review is to corroborate information regarding both the extraction and application of chitin, chitosan, and nanochitin, including new developments since the publication of those prior review articles.

## 2. Extraction Methods

Crustacean shells are largely composed of three components: Calcium carbonate, proteins, and chitin [22]. The extraction of chitin requires separation of chitin from the other parts of the shell. Extraction techniques can be broadly described as either chemical or biological methods. This section contains descriptions of chitin extraction methods, including specific developments, and culminates in a comparison of the benefits and drawbacks of each.

### 2.1. Chemical Methods

Chemical extraction of chitin usually involves two steps: Demineralization and deproteination. The order of these steps does not matter in most cases. Often, demineralization is performed before deproteination to increase the surface area for deproteination. Since demineralization is usually performed by exposing crustacean shells to acidic conditions and deproteination is performed by exposing the shell to alkaline conditions, this is referred to as the acid-alkali method of chitin extraction [2,5,11]. The acid-alkali method is the method most common in industrial extraction due to the low cost of reagents and lack of specialized equipment required for reactions [23]. Concerns about the release of toxic acid and alkaline waste into the environment have prompted research into alternatives. Recent developments include the use of ionic liquids, deep eutectic solvents, and other novel processes with the aim of green chitin extraction [12,24,25].

#### 2.1.1. Demineralization

Chemical demineralization typically involves exposing crustacean shells to acidic conditions to dissolve shell minerals that are then separated from the residual shell solids. Both mineral and organic acids have been employed for demineralization; hydrochloric acid (HCl) is the most used, though concerns about environmental toxicity have prompted a turn toward organic acids such as citric, acetic, and lactic acids [11,26]. Demineralization is usually performed at room temperature over a period of 2–3 h, though there has been some indication that these reaction times are excessively long with little to no benefit over shorter ones [5,26].

Due to the simplicity of chemical demineralization, modifications are typically in the concentration and choice of acid. Acids must be able to dissolve calcium carbonate, the principal shell mineral, and ideally produce water-soluble salts that can be easily separated from the shell remnants. Modifications in the demineralization process have also included using multiple treatments with acid. Pohling et al. performed a two-step demineralization with citric acid, washing the ground shrimp shells between treatments [27]. Despite the poor solubility of calcium citrate, they managed to remove shell minerals to an extent comparable to a typical one-step HCl demineralization [27]. Trung et al. also split the demineralization step into two stages by pre-treating whole shrimp shells with dilute hydrochloric acid for 12 and 24 h [28]. The intended use of this process was to store shells without odour, but it also reduced the ash content from approximately 22% to as low as 0.7% before the formal demineralization process; the following HCl demineralization further lowered the ash content of the shells [28]. Yang et al. bubbled carbon dioxide through deionized water to dissolve calcium carbonate, mimicking the natural process of speleogenesis through the prodution of carbonic acid [23]. Increasing the partial pressure of carbon dioxide allowed for removal of close to 100% of shell minerals, though large volumes of water were required due to the poor solubility of the calcium bicarbonate produced by the reaction [23]. Demineralization processes and results are summarized in Table 1.

#### 2.1.2. Deproteination

Chemical deproteination is the process by which chitin is separated from the other organic shell components. The most common method for this is to use strong bases and high temperatures to render the proteins water-soluble and wash them from the chitin. Sodium hydroxide (NaOH) is the most common base used for deproteination due to its low cost [5,11,23]. Reaction temperatures are typically between 65 °C and 100 °C, though some studies perform reactions at room temperature and some at temperatures above 100 °C [5,11,23,27]. Concerns about polluting the environment with high concentrations of sodium ions have directed researchers to alternative bases with less toxic cations (e.g., potassium hydroxide) or other deproteination methodologies [11]. Inspired by the ability of pressure cookers to tenderize proteins in meat, Yang et al. replaced reactions with strong bases with hot water in a pressure vessel [23]. Chitin produced through this method possessed a lower molecular weight than that produced through a normal acid/alkali method but were similar in terms of morphology and purity [23].

Borić et al. did away with aqueous reaction media entirely, instead looking to dielectric barrier discharge plasma (DBD plasma) to deproteinate chitin [24,25]. A preliminary work with unprocessed shrimp shells described up to a 42% reduction in protein content in 6 min [24]. Further research put shrimp shells through the DBD plasma apparatus after demineralization. The DBD plasma both stripped away any residual mineral content and approximately 90% of proteins contained within the shells [25]. The main advantage of the DBD plasma method over other deproteination methods is that the by-products were carbon monoxide, carbon dioxide, hydrogen, and nitrogen—all gaseous; the only solvent waste collected was the demineralization effluent [25]. Table 2 summarizes results from chemical deproteination.

#### 2.1.3. Ionic Liquids and Deep Eutectic Acids

Another method of chitin extraction is through solvation in ionic liquids (ILs). Ionic liquids are salts with melting points below 100 °C and are composed of a large organic cation and a smaller anion that is either organic or inorganic. They are attractive as “green” solvents due to their low vapour pressure, thermal stability, low flammability, and potential for recycling after use [12,34].

Ionic liquids have drawn attention for their ability to dissolve biomass, including polysaccharides with poor solubility [12]. Chitin extraction through ionic liquids involves dissolving crustacean shells in an ionic liquid and precipitating the chitin via an antisolvent, a reagent added to render the chitin insoluble [34]. Dissolution can involve temperatures above 100 °C, depending on the ionic liquid [12,34]. Common antisolvents include methanol, ethanol, and aqueous solutions; aqueous acids carry the benefit of aiding in demineralization, potentially yielding chitin with less contamination [34,35,36,37,38,39]. Ionic liquids dissolve chitin by inserting anions that disrupt the hydrogen bond network between and within chitin chains [39]. Factors influencing the ability for ionic liquids to dissolve chitin include the crystallinity, molecular weight, and DD of the chitin and the identity of the ionic liquid [34,39]. High crystallinity or high molecular weight of a chitin sample inhibit solvation [39]. Chitin solubility improves with higher DD values, particularly when the DD is 50% or higher [39]. Anions that act as hydrogen bond acceptors show greater ability to dissolve chitin, though this is also dependent on interactions with the cation [34,39].

Shimo et al. noted that ethylenediamine could enter chitin crystals and join the hydrogen bond network within [40]. Preliminary tests indicated that the addition of ethylenediamine to ionic liquids allowed for the dissolution of chitin at room temperature [40]. Tetraalkylammonium hydroxides may be an exception, as several have demonstrated the ability to dissolve chitin at room temperature [40,41].

Many ionic liquids have been demonstrated to dissolve chitin and could potentially be directed toward its extraction of biomass, including tetraalkylammonium hydroxides and dialkylimidazolium-based ionic liquids [12,39,41]. Tolesa et al. determined that ammonium-based ionic liquids diisopropylethylammonium acetate ([DIPEA][Ac]), diisopropylethylammonium propanoate ([DIPEA][P]), and dimethylbutylammonium acetate ([DMBA][Ac]) were all applicable in the dissolution of shrimp shells. Of these, [DIPEA][Ac] produced the highest yield of chitin after regeneration with aqueous citric acid [37]. Wineinger et al. extracted chitin from shrimp shells using 1-ethyl-3-methylimidazolium acetate ([EMIM][OAc]) to dissolve the shells and deionized water as an antisolvent [36]. Interested in replacing the complicated synthesis of [EMIM][OAc] with the simpler synthesis of an ionic liquid with a halide anion, Setoguchi et al. attempted the extraction of chitin from crab shells with 1-allyl-3-methylimidazolium bromide ([AMIM][Br]). The extraction was successful after regeneration with citric acid [38].

Criticisms have been levied at the “green” label associated with ionic liquids due to their toxicity and poor biodegradability [42]. Deep Eutectic Solvents (DESs) were developed as alternatives to ionic liquids without moisture sensitivity and with biodegradability in mind [43]. DESs are composed of a hydrogen bond donor and acceptor pair, allowing for similar solvation properties to ionic liquids. Early DESs contained metal ions [43]. In pursuit of lower toxicity, natural deep eutectic solvents (NADESs) were developed; NADESs are composed of primary metabolites and offer a lower-cost, biodegradable option for the extraction of biomass [42,44,45]. Among NADESs used for chitin extraction, choline chloride (ChCl)/organic acid combinations are the most common due to their low toxicity and ability to remove calcium carbonate; betaine is another common hydrogen bond acceptor, and hydrogen bond donors include organic molecules such as glycerol, ethylene glycol, and urea [12,13,46,47]. Saravana et al. tested deep eutectic solvents composed of choline chloride and various other components such as organic acids and alcohols for their ability to extract chitin from shells of the shrimp species *Marsupenaeus japonicus* [31]. Of the tested deep eutectic solvents, ChCl-malonic acid at a 1:2 ratio was deemed the best for chitin extraction. Higher yields were obtained with the NADES compared to an acid/alkali method, though the acid/alkali method was more effective at removing shell minerals and proteins [31]. To work around the relatively limited ability of NADESs to remove proteins shell minerals, Rodrigues et al. added water to the NADES/chitin solution after the extraction step and stirred for 30 min [46]. This both precipitated the chitin out of the NADES solution and generated an acidic environment to facilitate the removal of shell minerals and hydrolysis of proteins [46]. Adding a decolouration step with hydrogen peroxide produced chitin with up to 98% purity with one third of the losses associated with the acid/alkali method [46]. Use of microwave radiation has also been indicated to improve the demineralization and deproteination capabilities of DESs; in a study by Huang et al., it was found that more than 99% of minerals were removed from shrimp shells after 9 min of irradiation and that deproteination improved with a higher NADES/shell ratio up to the maximum 20:1 [48]. Similar effects were observed by Zhao et al., though no significant difference was observed in the chitin product after 7 min of microwave irradiation [49]. This study also included a demineralization step with citric acid before solvation in DESs to produce chitin of similar purity to an acid/alkali method with a higher yield [49].

NADESs can be composed of more than two components. Wang et al. tested a range of binary and ternary NADESs incorporating molecules similar to chitin, e.g., *N*-acetyl-D-glucosamine and D-gluconic acid, for their ability to extract chitin from the shells of snow crabs [33]. They determined that, of the tested NADESs, ChCl/*N-*acetyl-D-glucosamine/formic acid at a 1:0.6:1.4 ratio produced chitin with a higher yield and comparable purity to an acid/alkali method [33]. Other tested NADESs had some combination of low yield and low purity compared to the acid/alkali test [33]. The NADES selected as best produced chitin with a molecular weight more than triple that extracted through the acid/alkali method [33]. The NADES proved to be recyclable, though with a slight reduction in the purity of extracted chitin with each regeneration of NADES [33]. Bisht et al. corroborated the recyclability of DESs, with only approximately 5% loss of chitin weight with each reuse [47]. It was noted that the DESs became discoloured after three extractions, despite cleaning the solvents of protein and mineral waste with absolute ethanol between uses [47]. DESs can be recycled without flushing contaminants with other solvents, though accumulated protein and mineral waste can render the DES too viscous to effectively use as a solvent [48]. The viscosity of DESs, even those untainted by waste materials, render large-scale chitin extraction difficult. Table 3 summarizes some results of chitin extraction with ionic liquids and DESs.

### 2.2. Biological Methods

Biological methods of chitin extraction can be classified as either enzymatic methods or fermentation methods. As the names imply, the former uses enzymes to break down crustacean shells and the latter uses bacteria to digest the shells until only chitin remains [2,10,11]. In general, biological methods produce chitin with a high molecular weight and low DD but struggle to completely demineralize and deproteinate chitin [11,29].

#### 2.2.1. Enzymatic Methods

Enzymatic methods describe the use of proteinases to remove shell proteins and isolate chitin. Proteinases used in chitin extraction are commonly sourced from bacteria or the entrails of marine life [11,50]. Enzymatic methods exploit the specificity of enzymes and mild reaction conditions (commonly 25–59 °C) to remove proteins with minimal deacetylation and damage to the chitin chain [11,13,51]. Reaction times are similar to those seen in chemical deproteination: Typically on the scale of 2–8 h, though some reports allow reactions to continue up to 24 h [13]. Even with longer reaction times, it is rare for deproteination to exceed 90% through enzymatic methods; this is attributed to the loss of active sites to which enzymes can bind as proteins are removed from chitin [11,13,50,51,52]. Hamdi et al. reported that this limitation persists even when the optimum enzyme function is under alkaline (pH 8.0) conditions [50]. Cost is also a limiting factor, particularly when scaling up chitin production is considered; proteinases are costly compared to the bases used in chemical deproteination [11,51]. Despite the high cost, the supernatant produced through enzymatic extraction of chitin contains amino acids and is valuable as a nutritional resource [10,50].

#### 2.2.2. Fermentation Methods

Fermentation methods replace enzymes with bacterial cultures, decreasing costs when scaling up reactions. Unlike enzymatic extraction, fermentation methods can incorporate demineralization through the use of acid-producing bacteria; lactic acid-producing cultures are commonly used for this purpose [10,11,13]. Lactic acid fermentation can be sufficient for the complete extraction of chitin [53]. Some species of lactic acid-producing bacteria possess lackluster deproteination capabilities; fermentation with protease-producing cultures can follow to improve the purity of produced chitin [54,55,56]. Protease-producing bacteria alone rarely provide adequate demineralization and often require an additional demineralization step [55,56,57,58].

Fermentation methods of chitin extraction require the preparation of media to support culture growth. The medium may be a complicated mixture of reagents to achieve optimal chitin extraction [58]. Independent of optimization, all media require a source of carbon for the bacteria to convert into acid; glucose is a common selection for small-scale experiments [10,13]. To lower costs and exploit multiple waste streams, Tan et al. experimented with using the autoclaved waste peels and pulps of ten different fruits as the carbon source for fermentation [56]. Of the tested fruit waste, the pulp of red grapes proved to be the best carbon source for extraction of chitin, yielding 12.2% of the mass of the initial shrimp shells and producing chitin with lower protein and mineral contents than that produced with other fruit waste [56].

As with the enzymatic methods, the supernatant generated by fermentation carries valuable amino acids [58]. Biological extraction processes are summarized in Table 4. The largest drawback to fermentation methods are the long fermentation times. Experiments tend to last three or more days; fermentation times of a week are common [10,13].

### 2.3. Comparison of Extraction Methods

On average, the comparatively low costs of acids and bases required for acid/alkali extraction of chitin make it among the cheapest methods. Acid/alkali extraction has short reaction times, does not require highly specialized reactors, and effectively removes proteins and minerals to yield high purity chitin [11,13]. Even when low-toxicity organic acids are used for demineralization, the alkaline residue following deproteination is toxic in the environment; the intense reaction conditions also can lead to accidental deacetylation and chain degradation, which lowers the molecular weight and yield of produced chitin [11,13,59]. Ionic liquids solve the issues of low molecular weight and unwanted deacetylation but come at increased cost, though some of the cost is mitigated in the recyclability of ionic liquids. Ionic liquids are still toxic in the event of environmental release [12,34]. Deep eutectic solvents share the benefits of ionic liquids at a lower cost and lower environmental toxicity, though costs still exceed those of acid/alkali extraction and the high viscosity of DESs frustrate attempts to increase the scale of chitin extraction [12,59].

Biological methods carry the advantage of negligible environmental toxicity and high molecular weight products, though are far more costly than the acid/alkali method and struggle to completely demineralize and deproteinate chitin [11,29]. Enzymatic methods require a separate demineralization step, often performed with the same chemicals as in the acid/alkali method, and scale poorly due to the cost of enzymes [11,51]. Fermentation resolves the issues with scaling inherent to enzymatic methods but require specialized reactors and preparation of reaction media to fully take advantage of the cultures [10,11,13,29]. Biosecurity can also be a concern when attempting to import bacterial strains [11].

**Figure 2:**
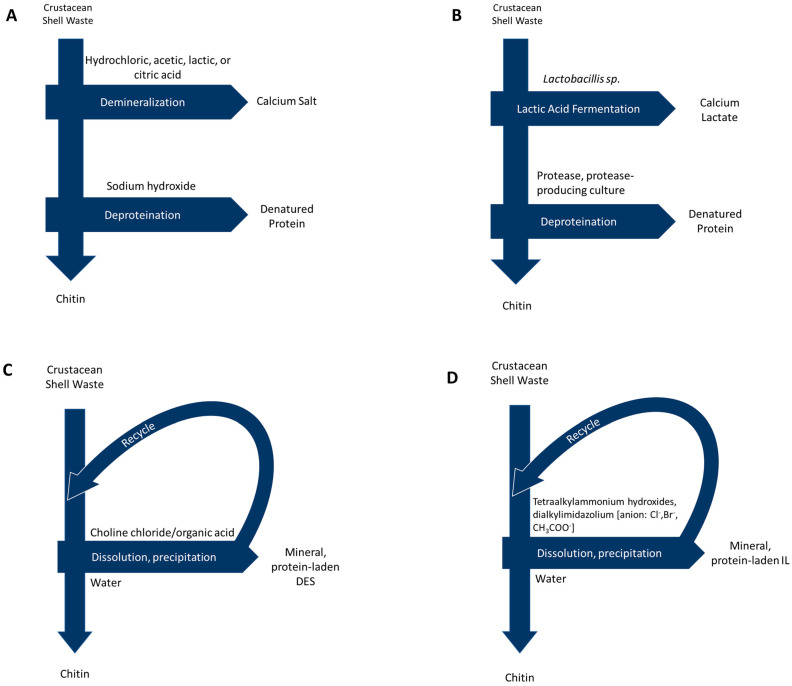
Process diagrams for chitin extraction via: (**A**) Acid/alkali methods (**B**) Biological methods (**C**) Deep eutectic solvents and (**D**) Ionic liquids.

Representations of extraction methods, including by-products, waste products, and common reagents, are shown in Figure 2.

## 3. Deacetylation

Chitin can be converted into chitosan through deacetylation, wherein *N*-acetyl groups are converted into amine. The traditional chemical approach is to expose chitin to concentrated alkaline solutions at temperatures usually above 100 °C [5,11,60]. As with deproteination, sodium hydroxide is a common choice for deacetylation, though at much higher concentrations; up to 50% sodium hydroxide is common, and some reports use concentrations as high as 70% [5,11]. The caustic conditions of chemical deacetylation can lead to reductions in the molecular weight of products [5,11,60]. Deacetylation reactions tend to last between 30 min and 5 h; some reactions last up to 24 h [5,11,61]. Longer reaction times, higher temperatures, and higher alkali concentrations correspond to higher degrees of deacetylation at the cost of molecular weight [5,61].

The biological method for deacetylation is through the enzyme chitin deacetylase, sourced from fungi, and is similar in execution and limitations to enzymatic methods of chitin extraction [11,54]. This method is rare, as the enzymes are not commercially available and those extracted from biomass suffer from low activity [41,62,63].

Several ionic liquids improve the activity of chitin deacetylase. Aspras et al. tested the effects of twelve ionic liquids on chitin deacetylase; of the tested ionic liquids, 1-allyl-3-methylimidazolium chloride ([Amim][Cl]) increased the function of deacetylase the most [62]. Other ionic liquids containing the chloride anion decreased the enzyme activity [62]. All tested ionic liquids containing the bromide anion increased enzyme activity [62]. Further work with butyl-3-methylimidazolium bromide ([Bmim][Br]) found that the ionic liquid increased deacetylase activity at low concentrations and inhibited activity at higher concentrations (above 1 mg/mL in solution with chitin deacetylase) [63]. Ma et al. found that tetrabutylammonium hydroxide also increased deacetylase activity, suggesting that the promotional effect is not limited to imidazolium-based ionic liquids [41].

**Figure 3:**
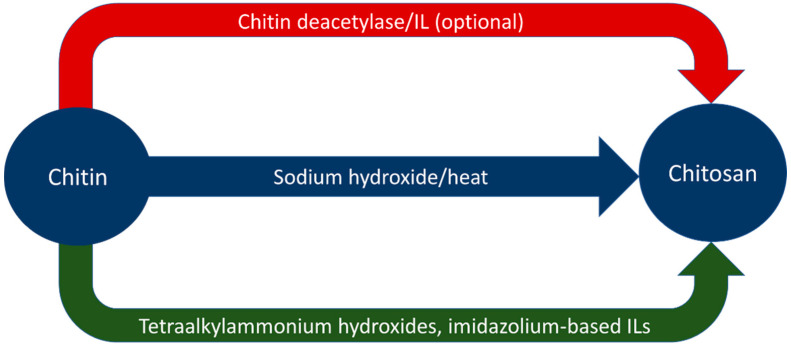
Graphical representation of deacetylation processes.

Ionic liquids have also demonstrated use in increasing the degree of deacetylation of chitosan. Ishii et al. dissolved chitosan in 1-butyl-3-methylimidazolium acetate (BMIMOAc) and heated the solution to 100 °C for 2 h under a nitrogen atmosphere [64]. This increased the degree of deacetylation in the chitosan from 77.3% to 86.7% with only acetic acid as a by-product [64]. The BMIMOAc was recovered, indicating that the solvent could be recycled for further use [64]. Aqueous tetraalkylammonium hydroxide solutions have indicated the ability to deacetylate dissolved chitin at ambient temperatures, though the experiment performed by Shimo et al. required two weeks to do so [40]. Table 5 summarizes the results from both alkali and ionic liquid deacetylation. Figure 3 graphically represents routes of deacetylation to yield chitosan from chitin.

## 4. Nanochitin Synthesis

**Figure 4:**
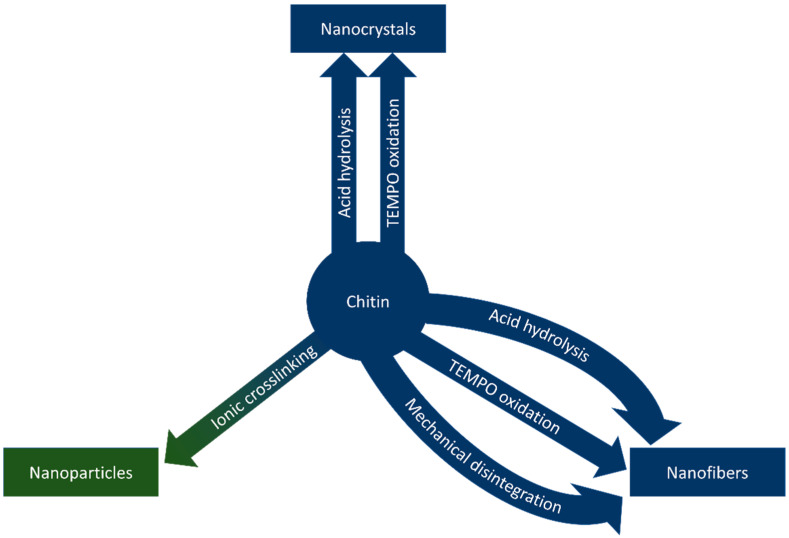
Methods to synthesize chitin nanowhiskers, nanofibers, and nanoparticles.

Nanochitin is a term that refers to chitin structures on the nanoscale. The morphology of chitin nanostructures varies based on the source and method of extraction, typically falling into the categories of nanofibers, nanocrystals, and nanoparticles, based on the morphology of the nanostructure [14]. Nanofibers and nanocrystals share a similar morphology; both are long, crystalline rods with high aspect ratios. Nanofibers differ from nanocrystals in length; nanocrystals are up to hundreds of nanometres long, while the length of nanofibers can extend into microns [15]. Nanoparticles describe nanostructures that lack the crystallinity and aspect ratios of the other morphologies due to differences in synthetic methods [14]. This section of the review describes methods to synthesize nanochitin and is graphically summarized in Figure 4.

Nanochitin was first synthesized by Revol and Marchessault in 1993 by exposing chitin to 3 M HCl for up to 6 h to produce chitin nanocrystals [65]. This method has become the template for the acid hydrolysis method of nanochitin synthesis, one of the more common methods of synthesizing nanochitin [14,15,66]. In acid hydrolysis, strong acids in concentrations near 3 M remove the amorphous regions of chitin, leaving behind short, highly crystalline fibers [14,15,65]. Reaction temperatures near 100 °C facilitate dissolution of the amorphous regions and ultrasonication often follows to better disperse the fibers [14,15]. Acid concentration influences the morphology of produced nanochitin; lower concentrations lead to larger particle sizes and, at sufficiently low concentrations, can leave some amorphous regions intact to yield chitin nanofibers instead of nanocrystals [67]. Acidic deep eutectic solvents can replace strong acids for a recyclable alternative to minimize waste [68].

Oxidation reactions mediated by the 2,2,6,6-tetramethylpiperidine-1-oxyl radical (TEMPO) have also been used as a method of nanochitin synthesis. In these reactions, NaBr, TEMPO, and a co-oxidant (e.g., NaClO) in basic conditions (pH 10–11) selectively oxidize the chitin C6 hydroxyl group over the course of 48 h, rendering amorphous regions of the chitin water-soluble [69]. The insoluble crystalline parts are chitin nanocrystals. Larger amounts of co-oxidant in the reaction yield shorter crystal lengths as more sites on the chitin chain are oxidized and dissolve [14,15,69]. TEMPO-mediated oxidation reactions can be performed at room temperature, though reaction times are far longer than those of acid hydrolysis [14,15,69]. Long reaction times and comparatively high cost contribute to the more widespread use of acid hydrolysis for nanochitin synthesis.

To decrease the reaction time and energy consumption of nanochitin extraction, Fernández-Marín et al. employed a microwave-assisted extraction technique to synthesize chitin nanocrystals and nanofibers [70]. Chitin was immersed in 1–3 M hydrochloric acid and reacted in a microwave for 10–30 min [70]. Yields and morphologies from microwave-assisted extraction were reported to be similar to those of TEMPO oxidation, traditional acid hydrolysis, and mechanical methods despite shorter reaction times [70]. This method was limited primarily by the requirement of a microwave reactor.

Mechanical disintegration typically produces chitin nanofibers rather than nanocrystals [15]. Mechanical methods can include the use of blenders, grinders, homogenizers, or microfluidizers and require only mildly acidic conditions [15,71]. Ultrasonication can yield chitin nanofibers in neutral pH conditions, though the process is facilitated by surface ionization methods such as partial deacetylation or oxidation [72]. These mild methods lack the ability to fully break down the amorphous regions to yield nanocrystals [15,71]. Partial deacetylation of chitin before mechanical disintegration yielded Fan et al. nanocrystals instead of nanofibers; this was attributed to the improved solubility of deacetylated chitin and electrostatic repulsion induced by cationic charges along the surface of the crystals [73].

Chitin nanoparticles also require sufficiently deacetylated chitin for synthesis, as they require cross-linking through amine groups not found in acetylated chitin [14]. In general, partially deacetylated or chemically modified chitin is dissolved in the presence of an ionic cross-linking agent (e.g., tripolyphosphate or iron (iii) chloride) [14,74]. Following cross-linking, the amorphous nanoparticles crash out of solution [74]. Synthetic methods for nanochitin are summarized in Table 6.

## 5. Applications

**Figure 5:**
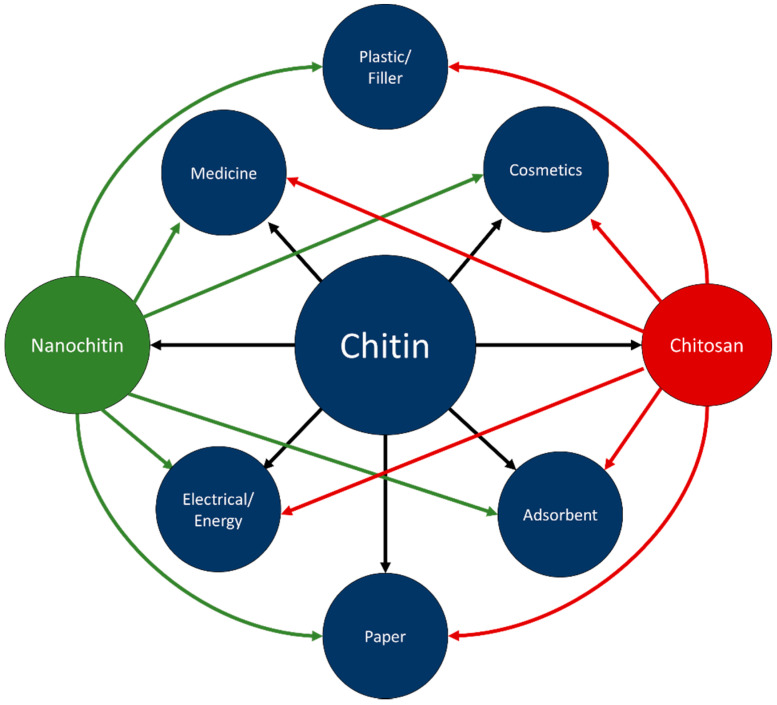
Connections between chitin, chitosan, nanochitin, and applications.

**Figure 6:**
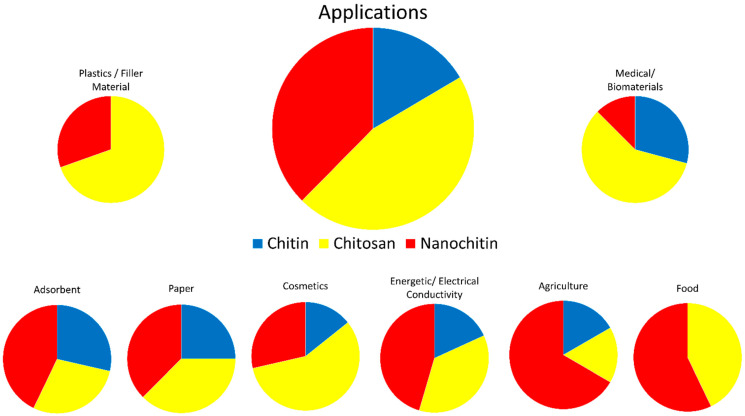
Estimated proportions of chitin, chitosan, and nanochitin used in various fields.

Chitin is inert in the digestive tracts of mammals, has low toxicity, and is biodegradable. In part due to these properties, chitin has found use in a wide range of fields, including cosmetics, paper making, wastewater treatment, wound dressing, and tissue engineering [3]. Chitosan has been researched for use in similar fields; improved solubility over chitin has led to chitosan also finding applications in plastics [3,6]. Figure 5 visually represents some of the applicable fields of chitin, chitosan, and nanochitin. Figure 6 graphically displays the proportion of each material for each field of application, estimated by the number of citations in each section of this review.

### 5.1. Filler Material/Plastics

Strong hydrogen bonds between chitin chains limit the solubility of chitin. Poor solubility has restricted the applications of chitin. By comparison, chitosan is soluble in mild acidic conditions, e.g., 1% acetic acid solutions; as a result, the bulk of application-side research has been devoted to chitosan rather than chitin, particularly regarding thin films [1,2,3,4]. Chitosan thin films are often synthesized through a simple solution-casting process. Briefly, chitosan solutions are cast onto a plate and the solvent is evaporated away, leaving behind a thin film. Chitosan thin films tend to be brittle; the inelasticity is attributed to hydrogen bonds between chitosan chains [78].

The properties of chitosan-based plastics can be manipulated with the addition of other materials. The addition of plasticizers such as glycerol or castor oil interrupt the hydrogen bonds in chitosan films, rendering the films more flexible and elastic [78]. When identifying the effects of additives on the mechanical properties of film, it is common to compare the tensile strength and elongation at break. Starches from various plant sources have been demonstrated to decrease the tensile strength of chitosan films and increase the elongation at break; these effects were observed independent of the addition of plasticizers [79,80,81]. Liquid smoke had a similar effect on the mechanical properties of cellulose/glycerol/chitosan plastics. Unlike the starches, liquid smoke slowed the biodegradation of the thin films [82]. Choo et al. observed a reduction in tensile strength and an increase in elongation at break with the addition of essential oils to chitosan/acetylated starch films. Increasing concentrations of essential oils in the films were also correlated to increased antimicrobial properties [83]. Essential oils do not universally manipulate the mechanical properties in this way; Castro et al. observed no statistically significant change in tensile strength with the addition of tea tree essential oil (TTEO) to chitosan/polyvinyl alcohol films up to 1.5% (*w/w*) concentration [84].

Conversely, chitosan as an additive to other plastics has been demonstrated to increase the tensile strength and add antimicrobial properties where none existed prior [82,85,86,87]. Phosphorylated cellulose nanocrystals and microcrystals increased the tensile strength and antimicrobial properties of chitosan films and improved biodegradability [88]. The addition of cellulose nanocrystals also increased the thermal stability of the films [88]. Kongkaorophtham et al. modified chitosan nanoparticles with organic polymers deoxycholic acid, poly(stearyl methacrylate), and poly(ethylene glycol) methyl ether methacrylate for use as an additive to polylactic acid films [89]. Addition of any of these modified nanoparticles improved the antimicrobial properties and tensile strength of the films and decreased the elongation at break [89]. The addition of metal oxides during the formation of chitosan films can improve the antimicrobial properties of the films, as demonstrated by Hammi et al. [90]. Excessive addition of metal oxides led to films becoming brittle and cracking [90]. Additional work by Sapei et al. suggested that the addition of metal oxides can also influence the mechanical properties of chitosan plastics; they found that adding zinc oxide to chitosan-banana starch plastics improved the tensile strength of the materials and decreased both the elongation and water uptake of the plastics [91].

The interest in chitosan plastics is in part derived from their biodegradability. Biodegradability renders chitosan plastics favourable over petroleum-based alternatives that persist in the environment, particularly for single-use applications. Plastics containing chitosan have been investigated as edible films for food packaging [92]. Chitosan-based films have been demonstrated to improve the shelf-life of both meats and produce [92,93,94]. The improvement to shelf-life has been attributed to a combination of chitosan acting as an antioxidant and vapour barrier in addition to its antibacterial and antifungal properties [92,93,94]. Biodegradability favours formulations other than thin films; though most plastics research is devoted to thin films, 3D objects on the scale of chess pieces have been constructed from chitosan via injection molding [95]. On that scale, changes in volume due to solvent evaporation became a concern; to minimize changes in volume during the evaporation process, Fernandez and Ingber added wood flour to the chitosan solutions [95].

Nanochitin has also been incorporated into edible films for food preservation. Heidari et al. produced thin chitin nanofiber films and nanocomposites containing biodegradable polymers with chitin nanofibers as the base [96]. Nanofiber/polyvinyl alcohol, nanofiber/gelatin, and nanofiber/thermoplastic starch films showed improved tensile strength, and greater water vapour permeability compared to pure nanofiber films [96]. Addition of nanochitins improved the tensile strength of polymer films and introduced antifungal and antibacterial effects [72,97,98,99]. Tests comparing chitin nanofibers to chitin nanocrystals as fillers indicated that nanofibers yield higher tensile strengths and greater fungal growth inhibition that films with nanocrystals as fillers [100]. These properties are not limited to thin films; chitin nanowhiskers cross-linked with chitosan via isocyanate hexamethylene-1,6-di-(aminocarboxysulfonate) (HDS) produced hydrogels with up to 80× greater tensile strength compared to neat chitosan hydrogels, though the swelling capacity was reduced by up to approximately 90% with the addition of nanowhiskers [76]. Comparison among plastic formulations is drawn through summary of their mechanical properties in Table 7.

### 5.2. Medical/Biomaterials

Chitin and chitosan are non-toxic and biodegradable, making them attractive for use in medicine as biomaterials. Both have been extensively researched and continue to be researched for their use in applications such as tissue engineering, drug delivery, and hemostatic dressings [3,16,17,101,102]. The interest shown in chitin and chitosan has carried over, to nanostructures; nanowhiskers, nanofibers, and nanoparticles continue to be investigated for biomedical applications, the latter of which drawing focus particularly for use in drug delivery [14,17,18]. Part of this interest is due to the antimicrobial properties of chitin and its derivatives; chitosan in particular is noted to effectively inhibit the growth of both gram-positive and gram-negative bacteria, a trait attributed to disruptions to bacterial membranes by the amine group present on the molecule [103]. Chitin demonstrates an inhibitory effect against gram-positive bacteria but may promote the growth of gram-negative bacteria [103]. The broader antimicrobial properties of chitosan and insolubility of chitin have led to broader use of chitosan in biomaterials [3,17,101].

Chitin and chitosan act as hemostats and have drawn interest in wound-healing applications; chitosan gauze is commercially available for this application. New research in this area seeks to devise composite materials to improve the hemostatic properties or exploit said properties to hasten regeneration in damaged tissue [101]. For example, He et al. prepared composite gauzes composed of chitosan and alginate and similar gauzes surface-modified with a catechol [104]. The former failed to match pristine chitosan gauze as a hemostat [104]. The gauze containing catechol, by comparison, reduced blood loss to 17% that of the pristine chitosan when compared in femoral artery and liver wounds in a rat model [104]. This composite dressing also reduced the secondary bleeding induced by removal of the dressing [104]. Pang et al. exploited the hemostatic and antimicrobial effects of chitin and chitosan through the synthesis of chitosan/dextran hydrogels reinforced with chitin nanowhiskers for use as a tissue adhesive [105]. The hydrogels displayed comparable mechanical properties to a commercially-available tissue adhesive while facilitating healing and staving off infection in a rat model [105].

Chitin and chitosan, both on the nanoscale and otherwise, have been incorporated into scaffolds for tissue engineering [3,19,72]. For the sake of brevity, this review will focus on bone engineering as an example in this field. There have been indications that hydroxyapatite-coated chitosan scaffolds mimic the extracellular matrix of bone [19,106]. Nanosilica, when added to a chitin scaffold, promoted the formation of hydroxyapatite in vitro, circumventing requirements to coat the chitin before use [107]. This concept was later extended by Christy et al. in their chitosan/poly(vinyl alcohol) scaffolds reinforced with zinc oxide and nano bioactive glass (nanosilica) [108]. The addition of zinc oxide was to delay biodegradation of the scaffold and improve antimicrobial activity without increasing the toxicity [109]. It was also noted that higher nano bioactive glass contents correlated to increased deposition of hydroxyapatite on the surface of the scaffold [108]. Karimipour-Fard et al. incorporated hydroxyapatite directly into the scaffold [109]. In their investigation of the benefits of incorporating chitin nanowhiskers into polycaprolactone/nano-hydroxyapatite scaffolds, it was noted that the addition of chitin nanowhiskers improved the scaffolds’ ability to attach to cells and the ability of osteoblasts to proliferate across the scaffold in vitro [108]. Chitin nanowhiskers also increased the rate of biodegradation without altering the pH of the experimental medium, further indicating use as a potential tissue engineering scaffold [109]. These scaffolds were produced through an additive deposition method; Chang et al. developed a bioink from water-soluble methacrylated glycol chitosan that could be loaded with osteoblasts in suspension for more traditional 3D printing methods [109,110]. The 3D printed scaffolds could be cured with visible light before use [110].

Many of the same properties that make chitin and derivatives attractive for tissue engineering lend themselves to drug delivery applications. Developments in ionic liquids and deep eutectic solvents have made chitin more accessible for use in drug delivery, particularly in the form of hydrogels [111]. As above, chitosan is broadly more attractive due to its solubility and more general anti-microbial effect [3,17]. Chitosan-based drug carriers have been developed for a wide range of delivery methods, including oral, nasal, intravenous, and transdermal pathways, among others [17]. Part of this flexibility is afforded by wide range of morphologies available to chitosan and the stability of chitosan conjugated to other common components to drug carriers (e.g., alginate) [18]. This conjugation capability makes chitosan attractive for drug delivery, as it can be functionalized to improve the targeted release of drugs [17,18]. Current research in drug delivery revolves around constructing non-toxic biodegradable carriers that release the encapsulated drugs at a specific location to limit toxicity to other regions of the body. An example of this philosophy is in the chitosan/alginate hydrogels prepared by Hoang et al. [112]. These hydrogels were prepared using a norbornene-tetrazine chemical cross-linker and designed for oral administration; despite the hydrophilic nature of the hydrogels, they were successfully loaded up to 44% (*w/w*) with hydrophobic ketoprofen [112]. The gels retained more than 90% of the loaded ketoprofen drug when immersed in simulated gastric acid (pH 2.2, 37 °C) and released over 80% in simulated intestinal fluid (pH 7.4, 37 °C) [112]. The hydrogels were observed to be non-toxic in vitro and completed biodegradation in four days [112]. Drug targeting isn’t limited to specific organs; drug carriers can be constructed to release their contents in response to specific conditions, such as the thiol-hyaluronic acid/chitosan nanocarriers developed by Xia et al. [113]. These carriers were stable under simulated physiological conditions in vitro and released the carried drug under the acidic and reduced environment surrounding SKBR3 breast cancer-derived cell line, with peak release at pH 4.5 and in the presence of 10 mM glutathione [113]. An additional benefit of these over prior research was the tunable charge on the nanocarriers, allowing for adjustment to better bind to a loaded drug; nanoparticles with higher thiol-hyaluronic acid to chitosan ratios bore a negative charge, whereas those with a higher chitosan content possessed a positive charge [113].

### 5.3. Adsorbents

Due to the presence of hydroxyl and nitrogenous groups, chitin and its derivatives can be used to adsorb metal ions in solution; the amine of chitosan, along with its solubility, makes it preferred over chitin for this application [3,16,72,114]. The adsorption of metal ions onto chitin or chitosan is a spontaneous process, leading to the development of chitin-based adsorbents for removal of heavy metals from water [114,115]. This process is generally most effective under mild acidic conditions; under alkaline conditions, adsorption is inhibited by metals forming metal oxides, and at low pH it is inhibited through competition between metal ions and protons in solution [114,115]. Developments in this field prioritize the creation of biodegradable adsorbents that can be easily separated from water following metal complexation. These adsorbents can be as simple as chitin nanofibers or chitosan nanoparticles; Siahkamari et al. compared chitin nanofibers and chitosan nanoparticles for their ability to complex Pb(II) ions in solution [116]. Their chitosan nanoparticles outperformed their chitin nanofibers, adsorbing lead ions at a rate of over 94 mg per gram of nanoparticles compared to over 60 mg per gram of nanofibers [116]. This was attributed to stronger interactions between Pb(II) and the chitosan amine compared to Pb(II) and the N-acetyl group found in the chitin nanofibers [116] Since both chitosan and nanochitin adsorb metal ions, Wu et al. experimented with chitosan/polyvinyl alcohol hydrogels containing ferromagnetic iron nanoparticles and nanochitin of undescribed morphology to form beads to adsorb Cu(II) ions [117]. The addition of magnetic nanoparticles and nanochitin improved the maximum Cu(II) adsorption capacity by approximately 50% and allowed for removal of the particles from the water via magnetic separation [117].

Metals are not the only pollutants chitin can separate from water. Yan et al. constructed superoleophobic membranes from chitin nanofibers to separate oil/water emulsions with >95% efficiency [118]. The membranes were reusable when cleaned with deionized water, showed no reduction in filtration after 30 cycles, and were stable under a wide range of temperatures and pH values [118]. Metal ions were also adsorbed by the membrane during emulsion separation, indicating practical application in treatment of contaminated waters [118]. Zhang et al. developed hydrogels from a combination of nanocellulose and partially-deacetylated nanochitin for the adsorption of arsenic and methylene blue dye [119]. The adsorbents showed no decrease in adsorption capacity following 5 reuse cycles and achieved adsorption capacities of 217 mg of arsenic per gram of adsorbent and 505 mg methylene blue per gram of adsorbent [119]. Congo Red and Red No.7 dyes have also been indicated to adsorb onto chitin, furthering the application of chitin in cleaning industrial waste streams [120].

While not directly related to industrial waste streams, methane gas can dissolve in bodies of water and be released into the atmosphere, where it contributes to the greenhouse effect. The hydrophilic nature of chitin normally renders it unable to adsorb methane; Xu et al. changed this by decorating nanochitin aerogels with silica, rendering them superhydrophobic and able to adsorb methane [121]. Adsorbed methane gas was also extracted from the aerogels, both after removal from the water and during adsorption [121].

### 5.4. Paper

Chitin and its derivatives have applications in the papermaking industry [3,16,122]. Chitin or chitosan added to pulp mixes improves the mechanical strength of the resultant paper; derivatives of chitin, such as phosphorylated chitin nanofibers, yield the same result [3,122,123]. Phosphorylated nanofibers also increased the paper’s resistance to heat and added self-extinguishing properties, permitting the use of paper under conditions where it would normally combust [123]. Chitin can also increase the mechanical strength when incorporated as a surface coating [16,122]. Coating paper with chitin or chitosan improves the printability of paper, introduces water vapour barrier properties, and introduce and antimicrobial effect; as with other applications, chitosan has the benefit of solubility and greater antimicrobial effects [122].

Traditional paper is made from cellulose. Paper can be made from chitin nanofibers; Kadokawa et al. dissolved chitin in a deep eutectic solvent, then regenerated the chitin through antisolvation with methanol, yielding a network of interwoven self-assembled chitin nanofibers reminiscent of paper [124]. Paper produced through this method was brittle; addition of benzylamine during regeneration inhibited the formation of crystalline structures in the paper and produced mechanical properties comparable to cellulose filter paper [124]. Chitin nanofiber papers can also be functionalized to modify their properties. For example, Naghdi et al. functionalized chitin nanofiber paper with nanoparticles for sensory applications [125]. Papers containing plasmonic gold or silver nanoparticles changed colour in the presence of Hg^2+^; papers containing dithizone or curcumin changed colour in contact with a range of heavy metal ions; carbon dots, quantum dots, or upconverting nanoparticles emitted light in the presence of S^2-^ or quercetin ions [125]. These nanoparticle-functionalized chitin nanofiber papers were developed to act as biosensors for use in paper-based blood assays [125].

### 5.5. Cosmetics

Chitin and chitosan act as antioxidants, protecting skin from oxidative damage when incorporated into cosmetics [126]. Due to the positive charge on chitosan, it can act as a humectant to maintain skin moisture or strengthen hair and act as a conditioner through interactions with hair keratin [126]. Suspensions of chitosan nanoparticles in guar gum have been indicated to reduce skin sebum levels in seborrhea patients [127]. Azimi et al. found that chitin nanofibrils electrosprayed onto cellulose films improved compatibility with skin cells and downregulated inflammatory cytokines, indicating potential applications for nanochitin in cosmetics [128].

### 5.6. Energetic/Electrical Conductivity

The use of chitin, chitosan, and nanochitin in energetic and electronic conductivity applications is part of a broader movement to produce electronic devices that are both sustainably-sourced and biodegradable; extensive research has been devoted to the role of chitin and derivatives in this field [72,129]. Chitin and chitosan have been investigated as components in graphene nanocomposites, demonstrating a wide range of energy-related applications including as parts of batteries, solar cells, and fuel cells [21]. Further work has included composites containing graphene oxide its conducting form, reduced graphene oxide; Dong et al. loaded demineralized shrimp cuticles with graphene oxide and heated the cuticles to both reduce the graphene oxide and carbonize the cuticles [130]. The result was a carbonized chitin nanofiber/reduced graphene oxide nanocomposite exhibiting electrical conductivity values in excess of 30 S/cm, indicating potential use as supercapacitors [130]. Aerogels composed of carbonized chitin nanofibrils loaded with cuprous oxide have also been indicated to act as supercapacitors, though they are held back by poor electrical conductivity and deformation with repeated charge/discharge cycles [131].

Conductivity has been observed in more than just graphene-containing nanocomposites; Wang et al. noted that chitosan and chitosan-NaCl films both transformed into a conductive state when temperatures exceeded 50 °C [132]. This did not damage the films; it was suggested that these films could be used in reusable early fire warning systems [132]. When the same research group went on to investigate graphene oxide-chitosan nanocomposites, they found that nanocoating thermal insulation improved the insulation’s thermal stability and inhibited mass transfers, rendering it safer in the event of a fire [133]. Exposure to an alcohol flame reduced the graphene oxide to the conducting form; carbonized chitosan protected the reduced graphene oxide, preventing thermal degradation and allowing for continued current to pass through the material, which in turn allowed for a continued alarm signal when connected to a fire alarm circuit [133].

### 5.7. Agriculture

Chitin, incorporated into fertilizers, acts as a source of nitrogen for plants [134]. Chitin can also be used as a seed coating to protect against fungi and bacteria [134]. The presence of chitin inhibits fungal growth and promotes plant immune response to fungal infection; chitin solvents are generally harsh to plants, and so chitosan or dispersions of nanochitin are more commonly used for this purpose [134,135,136,137]. Similar application of nanochitin to soil has been indicated to improve the yield and protein content of wheat [138,139]. In tobacco, chitin nanowhiskers shortened germination times and resulted in taller stems and larger leaves [134].

Chitin nanowhiskers conjugated to biopesticides have been demonstrated to improve the effectiveness of the pesticides versus insects with sucking mouthparts, such as aphids [140,141]. Isolated nanowhiskers displayed low dermal and oral toxicity in a rat model, though no comparison was made to those conjugated to pesticides [140].

### 5.8. Food

Chitin nanocrystals stabilized oil-in-water emulsions for up to one month [142]. Combination cellulose nanofibril/chitin nanocrystal-stabilized emulsions endured longer, lasting through six months of storage [143]. The nanomaterials acted as a coating to protect and isolate the oil droplets, preventing coalescence of the droplets and the production of a separate phase, thus stabilizing the emulsions [142,143]. The long-term stability offered by nanocellulose/nanochitin emulsifiers have rendered them valuable as sustainably sourced stabilizers for food-grade emulsions such as sauces, dressings, and dips [144].

Phlorotannins, a type of seaweed-based polyphenol, acted as a biopreservative when adsorbed onto the surface of chitin nanocrystals to form a phlorotannin-nanochitin complex [145]. When applied to sea bass fillets, they inhibited bacterial growth and prevented changes in biochemistry to extend the shelf-life for up to 3 days [145]. The complexes outperformed isolated phlorotannins and nanocrystals in terms of changes to fillet pH, secondary metabolite production, and microbial growth across the study [145]. Dragon fruit coated with chitosan ionically bonded to κ-carrageenan showed reduced post-harvest weight loss over thirty days compared to an uncoated control [92]. Chitosan films also increased the shelf-life of meats, in part due to their antimicrobial properties [93,94].

### 5.9. Other

Research into applications of chitin is an ongoing endeavour. Nanochitin in particular is a relatively new field for exploration, leading to a variety of applications that are difficult to fit under the prior topics. This section of the review takes note of some such nanochitin applications.

Kishimoto et al. coloured chitin nanofibers with reactive dyes without altering the morphology of the nanofibers [146]. The red, yellow, and blue fibers were mixed in suspension to yield secondary colours and added colour to chitin nanofiber/acrylic resins while maintaining transparency in the resins [146]. These coloured nanofibers were suggested for adding colour to materials that are difficult to dye [146].

In crustacean shells, chitin nanofibrils form hierarchical structures known as Bouligand spirals [1] (pp. 305–312). Chitin nanowhiskers are on a similar scale to the chitin nanofibers at the fundamental level of crustacean shells; as such, research has been devoted to developing biomimetic materials that emulate properties found in nature, including iridescence seen in the shells of beetles and mantis shrimps [147]. Partially-deacetylated chitin nanofiber hydrogels with integrated hydroxyapatite were hot-pressed to produce materials with a similar appearance, mechanical properties, and layered structure to nacre, the extremely hard material that composes pearls and the interior of some mollusc shells [148].

## 6. Prospective and Outlook

The vast quantities of crustacean shell waste produced by shellfish fisheries can act as a sustainable source of chitin. Chitin extraction methods used at scale either suffer from high cost, long reaction times, or production of toxic waste products. Improvement of the described methods vary by the method: Chemical methods should seek to reduce the toxic waste products, either through changes to less toxic reagents or through optimization of the concentration and volume to limit the risk of release; biological methods must improve the removal of residual mineral and protein content to justify higher costs and longer reaction times; ionic liquids must overcome the high cost and improve recycling to minimize waste; deep eutectic solvents share the struggles of ionic liquids, though at a lower cost. Deep eutectic solvents must instead contend with high viscosity acting as a barrier to large-scale chitin extraction.

Chitin deacetylation contends with many of the same issues as deproteination in an exaggerated form, as higher alkali concentrations, enzymes with low activity, or costly ionic liquids are used in reactions with higher temperatures or longer reaction times. This leads to higher energetic and reagent costs the must be decreased to lower the environmental impact and improve the economic opportunities of chitosan production. Similarly, the prevalence of acid hydrolysis in nanochitin synthesis creates a reflection of the issues of demineralization, again exacerbated by the higher concentrations of mineral acid that prohibit the replacement with organic acids. The production of nanocrystals needs particularly intense conditions to remove amorphous regions of chitin; replacing mineral acids with deep eutectic solvents, lowering energetic costs with microwave radiation, or substituting acid hydrolysis with methods such as TEMPO oxidation all lead to lower environmental impact, though each of these require more costly reagents or specific reactors. Decreasing the costs associated with these greener methods would aid in the sustainability of nanochitin production.

Chitin and its derivatives have a wide range of applications, including as biodegradable plastics, biomedical materials, and many others. Research continues to expand on and discover new uses for chitin and chitin derivatives. More specifically, steps are being taken to incorporate the desirable traits of chitin into materials while working around shortcomings such as insolubility and brittleness of chitin. Further work will develop materials containing chitin and other renewable materials with properties to meet everyday needs, allowing for the replacement of less sustainable materials.

## Figures and Tables

**Table 1:** Chemical methods of demineralization.

Method	Source Species	Concentration	Duration	Ash Content (%)	Reference
CO_2 (aq)_	Gray shrimp (*Crangon crangon*)	10 atm	2 h	<1	[23]
HCl	Marine shrimp (*Parapenaeopsis stylifera*)	1 M	24 h	0.01	[26]
HCl	Northern shrimp (*Pandalus borealis*)	31.45%	2 h	0.29 ± 0.05	[27]
Citric acid (x2)	Northern shrimp (*Pandalus borealis*)	50%	35 min/1 h	0.34 ± 0.12	[27]
HCl (after 12 h in 0.28 M HCl)	White shrimp (*Litopenaeus vannamei*)	0.80 M	12 h	0.1	[28]
Lactic acid	Red crab (*Chionoeetes japonicus*)	5%	5 days	6.0 ± 0.2	[29]
HCl	Rock lobster (*Jasis lalandii*)	31%	2 h	<2	[30]

**Table 2:** Chemical methods of deproteination.

Method	Source Species	Concentration	Temperature/ Duration	Chitin Yield/ Residual Protein	Reference
Pressurized hot water	Gray shrimp (*Crangon crangon*)	-	180 °C/1 h	-/4.7%	[23]
DBD plasma	Shrimp (Species unspecified)	-	-/6 min	-/58%	[24]
DBD plasma	Northern shrimp (*Pandalus borealis*)	-	-/2 × 6 min	17%/<10%	[25]
NaOH	Marine shrimp (*Parapenaeopsis stylifera*)	10%	90 °C/3 h	20%/<1%	[26]
NaOH	White shrimp (*Litopenaeus vannamei*)	0.68 M	Ambient/24 h	-/0.92–0.96%	[28]
NaOH	Rock lobster (*Jasis lalandii*)	5%	80–85 °C/2 × 30 min	24.0%/-	[30]
NaOH	Shrimp (*Marsupenaeus japonicus*)	10%	90 °C/3 h	16.08 ± 0.57%/ 1.13 ± 0.01%	[31]
NaOH	Lagoon crab (*Callinectes amnicola*)	2.39 M	70 ° C/2 h	19.36 %/-	[32]
NaOH	Snow crab (*Chionoectes opilo*)	10%	90 °C/3 h	58.7 ± 0.8% of available/-	[33]

**Table 3:** Ionic liquid and deep eutectic solvent methods of chitin extraction.

Method	Source Species	Reagent	Temperature/ Duration	Chitin Yield	Reference
DES	Shrimp (*Marsupenaeus japonicus*)	ChCl–lactic acid (1:2)	80 °C/2 h	29.20 ± 1.97%	[31]
		ChCl–ethylene glycol (1:2)		52.45 ± 2.01%	
		ChCl–urea (1:2)		50.54 ± 1.07%	
		ChCl–malonic acid (1:2)		23.86 ± 0.07%	
DES	Snow crab (*Chionoecetes opilio*)	ChCl–*N*-acetyl-D-glucosamine (2:1)	130 °C/3 h	90.6 ± 1.2% *	[33]
		ChCl–D-gluconic acid (1:2)		82.7 ± 0.7% *	
		Betaine–D-gluconic acid (1:2)		90.7 ± 1.6% *	
		ChCl–*N*-acetyl-D-glucosamine–formic acid(1:1:1)		88.2 ± 1.2% *	
		(1:0.6:1.4)		85.6 ± 2.4% *	
IL	White shrimp (*Litopenaeus vannamei*)	[EMIM][OAc]	80 °C/0.5 h	13–18%	[36]
IL	Shrimp (Species unspecified)	[DIPEA][Ac]	110 °C/18 h	11.1%	[37]
			110 °C/24 h	13.4%	
			110 °C/30 h	14.7%	
		[DIPEA][P]	110 °C/18 h	10.0%	
			110 °C/24 h	11.5%	
			110 °C/30 h	12.1%	
		[DMBA][Ac]	110 °C/18 h	10.9%	
			110 °C/24 h	12.2%	
			110 °C/30 h	13.7%	
IL	Red queen crab	[AMIM][Br]	80 °C/24 h	6.1%	[38]
			100 °C/24 h	7.5%	
			120 °C/24 h	12.6%	
DES	Red crayfish	ChCl–lactic acid (1:2)	115 °C/20 h	85 ± 1%	[47]
		Betaine–lactic acid (1:2)		85 ± 1%	

* Yield taken in terms of available chitin, not total mass.

**Table 4:** Biological methods of chitin extraction.

Bacterial Strain/Enzyme	Source Species	Carbon Source	Duration	Deproteination (%)/ Demineralization (%)/ Chitin Yield (%)	Reference
Protease from *P. segnis* viscera	Blue crab (*Portunus segnis*) Shrimp (*Penaeus kerathurus)*	-	3 h	84.69 ± 0.65/100 */ 19.06 ± 1.65 91.06 ± 1.40/100 */ 22.23 ± 0.94	[50]
Protease from *Streptomyces griseus*	Shrimp (*Litopenaeus vannamei*)	-	3 h	91.10/98.64 */-	[51]
*Pseudomonas* *aeruginosa*	Shrimp (*Penaeus merguiensis*)	Glucose	6 days	96.44 ± 0.72/ */ 23.23 ± 3.75	[53]
*Lactobacillus* *acidophilus*	Pacific white leg shrimp (*Litopenaeus vannamei*)	Glucose	3 days	76/90.7/7.7	[54]
*Lactobacillus rhamnoides,* *Bacillus* *amyloliquefaciens*	Shrimp (*Litopenaeus vannamei*)	Glucose	7 days	96.8/97.5/19.6	[55]
*Lactobacillus plantarum,* *Bacillus subtilis*	Shrimp (Species unspecified)	Glucose	5 days	-/-/10.0	[56]
		Red grape Pomade		-/-/12.2	
		White grape Pomade		-/-/11.8	
		Mango peel		-/-/11.0	
*Brevibacillus* *parabrevis*	Giant tiger shrimp	Shrimp head powder	4 h	95.91 ± 2.01/ */ 16.87 ± 3.03	[57]
*Bacillus cereus*	Shrimp (*Penaeus monodon)*	Shrimp shell powder	7 days	97.42 ± 0.28/53.76 ± 0.21 (90 *)/-	[58]

* Chemical demineralization step.

**Table 5:** Methods and results of chitin deacetylation.

Method	Reagent	Temperature/Duration	Chitosan Yield	DD	Reference
Alkali	50% NaOH	84.46 °C/ 187 min	-	84.2%	[32]
Alkali	40% NaOH	100 °C/12 h	-	93%	[37]
IL	[N_2,2,2,2_][OH]/water (1:7.5)	Ambient/ 2 weeks	-	91%	[40]
Alkaline	50% NaOH	120 °C/4 h	-	71.9%	[52]
Alkaline	50% NaOH	121 °C/ 30 min	50%	80%	[60]
Alkaline	50% KOH, ethanol, monoethyleneglycol	120 °C/24 h	75% (*Maia squinado*) 77% (*Homarus vulgaris*)	97% 90%	[61]
IL	[BMIMO][Ac]	100 °C/2 h	-	86%	[64]

**Table 6:** Methods and results of nanochitin synthesis.

Method	Product	Yield	Dimensions (Diameter/Length) (nm)	Reference
Acid hydrolysis	Nanocrystals	65%	6–8/50–300	[65]
Acid hydrolysis	Nanocrystals	87.5%	42–49/257–670	[68]
TEMPO-mediated oxidation	Nanocrystals	90%	8/340	[69]
Microwave irradiation	Nanocrystals	85.3 ± 0.37% (Lobster)	41.62 ± 10.92/ 314.74 ± 62.50	[70]
		79.92 ± 0.24% (shrimp)	42.16 ± 4.62/ 386.12 ± 47.49	
Mechanical disintegration	Nanofibers	-	3.6–3.9/1000–1500	[71]
Partial deacetylation, mechanical disintegration	Nanocrystals	85–90%	6.2 ± 1.1/250 ± 140	[73]
Dissolution, cross-linking	Nanoparticles	-	237–429 (diameter)	[74]
Acid hydrolysis	Nanocrystals	86%	18–40/200–560	[75]
Acid hydrolysis	Nanocrystals	55–60%	6–8/100–200	[76]
Acid hydrolysis	Nanocrystals	40%	20/300	[77]

**Table 7:** Mechanical properties of polymer films containing chitosan or nanochitin.

Composition	Tensile Strength (MPa)	Elongation at Break (%)	Young’s Modulus (MPa)	Reference
40/60 Chitosan/yellow pumpkin starch, 15% *v*/*v* castor oil	6.787 ± 0.274	13.451 ± 3.709	6.093	[80]
60/40 Chitosan/yellow pumpkin starch, 15% *v*/*v* castor oil	2.563 ± 1.055	7.285 ± 1.135	5.263	
Chitosan, 30% *v*/*v* glycerol	5	14	-	[81]
70/30 Chitosan/banana starch, 30% *v*/*v* glycerol	2.5	28		
50/50 Chitosan/cassava peel starch, 30% *v/v* glycerol,	90	35	-	[82]
50/50 Chitosan/cassava peel starch, 30% *v/v* glycerol, 1 mL liquid smoke	85	42		
50/50 Chitosan/cassava peel starch, 30% *v/v* glycerol, 2 mL liquid smoke	55	28		
Chitosan	13.5	56	-	[83]
Chitosan/1.0% *v/v* essential oil	12.5	22		
Chitosan/2.0% *v/v* essential oil	10.0	32		
Starch film	9.54 ± 0.84	51.01 ± 1.32	16.50 ± 1.10	[86]
Starch/1% *w/w* Chitosan nanoparticle	14.74 ± 16.7	46.19 ± 1.71	24.20 ± 1.04	
Starch/4% *w/w* Chitosan nanoparticle	24.91 ± 0.81	34.29 ± 1.69	47.11 ± 2.51	
Chitosan film	40	27	1200	[88]
Chitosan/3% *w/w* microcrystalline cellulose	43	20	1350	
Chitosan/3% *w/w* nanocrystalline cellulose	50	24	1400	
Polylactic acid film	46	5	-	[89]
PLA/2% *w/w* chitosan nanoparticle	33	6.5		
PLA/4% *w/w* chitosan nanoparticle	27	7.3		
PLA/10% *w/w* chitosan nanoparticle	23	6.3		
70/30 Chitosan/starch, 30% *v/v* glycerol	2	28	-	[91]
70/30 Chitosan/starch, 1% *w/w* ZnO, 30% *v/v* glycerol	30	10		
70/30 Chitosan/starch, 3% *w/w* ZnO, 30% *w/w* glycerol	35	8		
70/30 Chitosan/starch, 5% *w/w* ZnO, 30% *w/w* glycerol	17	7		
Starch	1.5	77	5	[97]
Starch/5% *w/w* chitin nanocrystals	2	35	25	
Starch/20% *w/w* chitin nanocrystals	3	18	60	
Starch/5% *w/w* chitin nanofibers	5	16	30	
Starch/20% *w/w* chitin nanofibers	10.5	3	400	
Maize starch	1.64 ± 0.11	175 ± 7.07	-	[99]
Maize starch/0.5% *w/w* chitin nanowhiskers	2.79 ± 0.08	176 ± 8.65		
Maize starch/1% *w/w* chitin nanowhiskers	3.69 ± 0.07	179 ± 7.07		
Maize starch/5% *w/w* chitin nanowhiskers	2.37 ± 0.04	111 ± 4.24		

## Data Availability

Not applicable.

## References

[1] Meyers M.A., Chen P.Y. (2014). Biological Materials Science: Biological Materials, Bioinspired Materials, and Biomaterials.

[2] Hossin M.A., Al Shaqsi N.H.K., Al Jouby S.S.J.A., Al Sibani M.A. (2021). A review of polymeric chitin extraction, characterization, and applications. Arab. J. Geosci..

[3] Rinaudo M. (2006). Chitin and chitosan: Properties and applications. Prog. Polym. Sci..

[4] Shamshina J.L., Zavgorodnya O., Rogers R.D. (2018). Advances in Processing Chitin as a Promising Biomaterial from Ionic Liquids. Adv. Biochem. Eng. Biotechnol..

[5] No H.K., Meyers S.P. (2010). Preparation and Characterization of Chitin and Chitosan—A Review. J. Aquat. Food Prod. Technol..

[6] Ji J., Wang L., Yu H., Chen Y., Zhao Y., Zhang H., Amer W.A., Sun Y., Huang L., Saleem M. (2014). Chemical Modifications of Chitosan and Its Applications. Polym.-Plast. Technol. Eng..

[7] FAO (2020). The State of World Fisheries and Aquaculture 2020.

[8] Reboucas J.S.A., Oliveira F.P.S., Araujo A.C.S., Fouveia H.L., Latorres J.M., Martins V.G., Prentice C., Tesser M.B. (2021). Shellfish industrial waste reuse. Crit. Rev. Biotechnol..

[9] Yan N., Chen X. (2015). Sustainability: Don’t waste seafood waste. Nature.

[10] Arbia W., Arbia L., Adour L., Amrane A. (2012). Chitin Extraction from Crustacean Shells Using Biological Methods—A review. Food Technol. Biotechnol..

[11] Kou S., Peters L., Mucalo M. (2018). Chitosan: A review of sources and preparation methods. Int. J. Biol. Macromol..

[12] Morais E.S., da Costa Lopes A.M., Freire M.G., Freire C.S.R., Coutinho J.A.P., Silvestre A.J.D. (2020). Use of Ionic Liquids and Deep Eutectic Solvents in Polysaccharides Dissolution and Extraction Processes towards Sustainable Biomass Valorization. Molecules.

[13] Mohan K., Ganesan A.R., Ezhilarasi P.N., Kondamareddy K.K., Rajan D.K., Sathishkumar P., Rajarajeswaran J., Conterno L. (2022). Green and eco-friendly approaches for the extraction of chitin and chitosan: A review. Carbohydr. Polym..

[14] Jin T., Liu T., Lam E., Moores A. (2021). Chitin and Chitosan on the nanoscale. Nanoscale Horizons.

[15] Salaberria A.M., Labidi J., Fernandes S.C.M. (2015). Different routes to turn chitin into stunning nano-objects. Eur. Polym. J..

[16] Kumar M.N.V.R. (2000). A review of chitin and chitosan applications. React. Funct. Polym..

[17] Parhi R. (2020). Drug delivery applications of chitin and chitosan: A review. Environ. Chem. Lett..

[18] Ali A., Ahmed S. (2018). A review on chitosan and its nanocomposites in drug delivery. Int. J. Biol. Macromol..

[19] Tao F., Cheng Y., Shi X., Zheng H., Du Y., Xiang W., Deng H. (2020). Applications of chitin and chitosan nanofibers in bone regenerative engineering. Carbohydr. Polym..

[20] Hamed I., Ozogul F., Regenstein J.M. (2016). Industrial applications of crustacean by-products (chitin, chitosan, and chitooligosaccharides): A review. Trends Food Sci. Technol..

[21] Ikram R., Jan B.M., Qadir M.A., Sidek A., Stylianakis M.M., Kenanakos G. (2021). Recent Advances in Chitin and Chitosan/Graphene-Based Bio-Nanocomposites for Energetic Applications. Polymers.

[22] Nagasawa H. (2012). The crustacean cuticle: Structure, composition and mineralization. Front. Biosci..

[23] Yang H., Gozaydn G., Nasaruddin R.R., Har J.R.G., Chen X., Wang X., Yan N. (2019). Toward the Shell Biorefinery: Processing Crustacean Shell Waste using Hot Water and Carbonic Acid. ACS Sustain. Chem. Eng..

[24] Boric M., Puliyalil H., Novak U., Lokozar B. (2018). An intensified atmospheric plasma-based process for the isolation of the chitin biopolymer from waste crustacean biomass. Green Chem..

[25] Boric M., Vicente F.A., Jurkovic D.L., Novak U., Likozar B. (2020). Chitin isolation from crustacean waste using a hybrid demineralization/DBD plasma process. Carbohydr. Polym..

[26] Percot A., Viton C., Domard A. (2003). Optimization of Chitin Extraction from Shrimp Shells. Biomacromolecules.

[27] Pohling J., Dave D., Liu Y., Murphy W., Trenholm S. (2022). Two-step demineralization of shrimp (*Pandalus borealis*) shells using citric acid: An environmentally friendly, safe cost-effective alternative to the traditional approach. Green Chem..

[28] Trung T.S., Tram L.H., Tan N.V., Hoa N.V., Minh N.C., Loc T.L., Stevens W.F. (2020). Improved method for production of chitin and chitosan from shrimp shells. Carbohydr. Res..

[29] Jung W.J.J., Jo G.H., Kuk J.H., Kim K.Y., Park R.D. (2005). Demineralization of Crab Shells by Chemical and Biological Treatments. Biotechnol. Bioprocess Eng..

[30] Blumberg R., Southall C.L., Van Rensburg N.J., Volckman O.B. (1951). The Rock Lobster: A Study of Chitin Production from Processing Wastes. J. Sci. Food Agric..

[31] Saravana P.S., Ho T.C., Chae S.J., Cho Y.J., Park J.S., Lee H.J., Chun B.S. (2018). Deep eutectic solvent-based extraction and fabrication of chitin films from crustacean waste. Carbohydr. Polym..

[32] Olafadehan O.A., Ajayi T.O., Amoo K.O. (2020). Optimum Conditions for Extraction of Chitin and Chitosan from *Callinectes amnicola* Shell Waste. Theor. Found. Chem. Eng..

[33] Wang Y., Yang Y., Wang R., Zhu Y., Yang P., Lin Z., Wang Z., Cong W. (2022). Efficient extraction of chitin from crustacean waste via a novel ternary natural deep eutectic solvents. Carbohydr. Polym..

[34] Roy J.C., Salaün F., Giraud S., Ferri A. (2017). Solubility of Chitin: Solvents, Solution Behaviors and Their Related Mechanisms in Solubility of Polysaccharides. IntechOpen.

[35] Tan X., Wang G., Zhong L., Xie F., Lan P., Chi B. (2021). Regeneration behavior of chitosan from ionic liquid using water and alcohols as anti-solvents. Int. J. Biol. Macromol..

[36] Wineinger H.B., Kelly A., Shamshina J.L., Rogers R.D. (2020). Farmed Jumbo shrimp molts: An ionic liquid strategy to increase chitin yield per animal while controlling molecular weight. Green Chem.

[37] Tolesa L.D., Gupta B.S., Lee M.J. (2019). Chitin and chitosan production from shrimp shells using ammonium-based ionic liquids. Int. J. Biol. Macromol..

[38] Setoguchi T., Kato T., Yamamoto K., Kadokawa J. (2012). Facile production of chitin from crab shells using ionic liquid and citric acid. Int. J. Biol. Macromol..

[39] Wang W.T., Zhu J., Wang X.L., Huang Y., Wang Y.Z. (2010). Dissolution behavior of chitin in ionic liquids. J. Macromol. Sci. Part B.

[40] Shimo M., Abe M., Ohno H. (2016). Functional comparison of polar ionic liquids and onium hydroxides for chitin dissolution and deacetylation to chitosan. Sustain. Chem. Eng..

[41] Ma Q., Gao X., Bi X., Han Q., Tu L., Yang Y., Shen Y., Wang M. (2020). Dissolution and deacetylation of chitin in ionic liquid tetrabutylammonium hydroxide and its cascade reaction in enzyme treatment for chitin recycling. Carbohydr. Polym..

[42] Smith E.L., Abbot A.P., Ryder K.S. (2014). Deep Eutectic Solvents (DESs) and Their Applications. Chem. Rev..

[43] Abbot A.P., Capper G., Davies D.L., Munro H.L., Rasheed R.K., Tambyrajah V. (2001). Preparation of novel, moisture-stable, Lewis-acidic ionic liquids containing quaternary ammonium salts with functional side chains. Chem. Commun..

[44] Paiva A., Craveiro R., Aroso I., Martins M., Reis R.L., Duarte A.R.C. (2014). Natural Deep Eutectic Solvents—Solvents for the 21st Century. ACS Sustain. Chem. Eng..

[45] Dai Y., van Spronsen J., Witkamp G.-J., Verpoorte R., Choi Y.H. (2013). Natural deep eutectic solvents as new potential media for green technology. Anal. Chim. Acta.

[46] Rodrigues L.A., Redovnikovic I.R., Duarte A.R.C., Matias A.A., Paiva A. (2021). Low-Phytotoxic Deep Eutectic Sysytems as Alternative Extraction Media for the Recovery of Chitin from Brown Crab Shells. ACS Omega.

[47] Bisht M., Macario I.P.E., Neves M.C., Pereira J.L., Pandey S., Rogers R.D., Coutinho J.A.P., Ventura S.P.M. (2021). Enhanced Dissolution of Chitin Using Acidic Deep Eutectic Solvents: A Sustainable and Simple Approach to Extract Chitin from Crayfish shell Wastes as Alternative Feedstocks. ACS Sustain. Chem. Eng..

[48] Huang W.C., Zhao D., Guo N., Xue C., Mao X. (2018). Green and Facile Production of Chitin from Crustacean Shells Using a Natural Deep Eutectic Solvent. J. Agric. Food Chem..

[49] Zhao D., Huang W.C., Guo N., Zhang S., Xue C., Mao X. (2019). Two-Step Separation of Chitin from Shrimp Shells Using Citric Acid and Deep Eutectic Solvents with the Assistance of Microwave. Polymers.

[50] Hamdi M., Hammami A., Hajji S., Jridi M., Nasri M., Nasri R. (2017). Chitin extraction from blue crab (*Porunis segnis*) and shrimp (*Penaeus kerathurus*) shells using digestive alkaline proteases from *P. segnis* viscera. Int. J. Biol. Macromol..

[51] Hongkulsup C., Khutoryanskiy V.V., Niranjan K. (2016). Enzyme assisted extraction of chitin from shrimp shells (*Litopenaeus vannamei*). J. Chem. Technol. Biotechnol..

[52] Sayari N., Sila A., Abdelmalek B.E., Abdallah R.B., Ellouz-Chaabouni S., Bougatef A., Balti R. (2016). Chitin and chitosan from the Norway lobster by-products: Antimicrobial and anti-proliferative activities. Int. J. Biol. Macromol..

[53] Sedaghat F., Yousefzadi M., Toiserkani H., Najafipour S. (2017). Bioconversion of shrimp waste Penaeus merguiensis using lactic acid fermentation: An alternative procedure for chemical extraction of chitin and chitosan. Int. J. Biol. Macromol..

[54] Tan J.S., Abbasiliasi S., Lee C.K., Phapugrangkul P. (2020). Chitin extraction from shrimp wastes by single step fermentation with Lactobacillus acidophilus FTDC3871 using response surface methodology. J. Food Process. Preserv..

[55] Liu Y., Xing R., Yang H., Liu S., Qin Y., Li K., Yu H., Li P. (2020). Chitin extraction from shrimp (*Litopenaeus vannamei*) shells by successive two-step fermentation with *Lactobaccilus rhamnoides* and *Bacillus amyloliquefaciens*. Int. J. Biol. Macromol..

[56] Tan Y.N., Lee P.P., Chen W.N. (2020). Microbial extraction of chitin from seafood waste using sugars derived from fruit waste-stream. AMB Express.

[57] Doan C.T., Tran T.N., Nguyen V.B., Vo T.P.K., Nguyen A.D., Wang S.L. (2019). Chitin extraction from shrimp waste by liquid fermentation using an alkaline protease-producing strain, *Brevibacillus parabrevis*. Int. J. Biol. Macromol..

[58] Cahyaningtyas H.A.A., Suyotha W., Cheirsilp B., Prihanto A.A., Yano S., Wakayama M. (2022). Optimization of protease production by *Baccilus cereus* HMRSC30 for simultaneous extraction of chitin from shrimp shell with value-added recovered products. Environ. Sci. Pollut. Res..

[59] Morgan K., Conway C., Faherty S., Quigley C. (2021). A Comparative Analysis of Conventional and Deep Eutectic Solvent (DES)-Mediated Strategies for the Extraction of Chitin from Marine Crustacean Shells. Molecules.

[60] Agarwal M., Agarwal M.K., Shrivastav N., Pandey S., Gaur P. (2018). A Simple and Effective Method for Preparation of Chitosan from Chitin. Int. J. Life Sci. Scienti. Res..

[61] Rhazi M., Desbrieres J., Tolaimate A., Alagui A., Vottero P. (2000). Investigation of different natural sources of chitin: Influence of the source and deacetylation process on the physiochemical characteristics of chitosan. Polym. Int..

[62] Aspras I., Kaminska M., Karzynski K., Kawka M., Jaworska M.M. (2016). The influence of selected ionic liquids on activity of chitin deacetylase. Chem. Process Eng..

[63] Aspras I., Jaworska M.M., Gorak A. (2017). Kinetics of chitin deacetylase activation by the ionic liquid [Bmim][Br]. J. Biotechnol..

[64] Ishii D., Ohashi C., Hayashi H. (2014). Facile enchancement of the deacetylation degree of chitosan by hydrothermal treatment in an imidazolium-based ionic liquid. Green Chem..

[65] Revol J.-F., Marchessault R.H. (1993). In vitro chiral nematic ordering of chitin crystallites. Int. J. Biol. Macromol..

[66] Kadokawa J. (2013). Preparation and Applications of Chitin Nanofibers/Nanowhiskers. Biopolymer Nanocomposites: Processing, Properties, and Applications.

[67] Joseph B., Sam R.M., Balakrishnan P., Maria H.J., Gopi S., Volova T., Fernandes S.C.M., Thomas S. (2020). Extraction of Nanochitin from Marine Resources and Fabrication of Polymer Nanocomposites: Recent Advances. Polymers.

[68] Yuan Y., Hong S., Lian H., Zhang K., Liimatainen H. (2020). Comparison of acidic deep eutectic solvents in production of chitin nanocrystals. Carbohydr. Polym..

[69] Fan Y., Saito T., Isogai A. (2008). Chitin Nanocrystals Prepared by TEMPO-mediated Oxidation of α-Chitin. Biomacromolecules.

[70] Fernandez-Marin R., Hernandez-Ramos F., Salaberria A.M., Andres M.A., Labidi J., Fernandes S.C.M. (2021). Eco-friendly isolation and characterization of nanochitin from different origins by microwave irradiation: Optimization using response surface methodology. Int. J. Biol. Macromol..

[71] Mushi N.E., Butchosa N., Salajkova M., Zhou Q., Berglund L.A. (2014). Nanostructured membranes based on native chitin nanofibers prepared by mild process. Carbohydr. Polym..

[72] Bai L., Liu L., Esquivel M., Tardy B.L., Huan S., Niu X., Liu S., Yang G., Fan Y., Rojas O.J. (2022). Nanochitin: Chemistry, Structure, Assembly, and Applications. Chem. Rev..

[73] Fan Y., Saito T., Isogai A. (2010). Individual chitin nano-whiskers prepared from partially deacetylated α-chitin by fibril surface cationization. Carbohydr. Polym..

[74] Geetha P., Sivaram A.J., Jayakumar R., Gopi Mohan C. (2016). Integration of in silico modeling, prediction by binding energy and experimental approach to study the amorphous chitin nanocarriers for cancer drug delivery. Carbohydr. Polym..

[75] Phongying S., Aiba S.I., Chirachanchai S. (2007). Direct chitosan nanoscaffold formation via chitin whiskers. Polym. J..

[76] Araki J., Yamanaka Y., Ohkawa K. (2012). Chitin-chitosan nanocomposite gels: Reinforcement of chitosan hydrogels with rod-like chitin nanowhiskers. Polym. J..

[77] Ma B., Qin A., Li X., Zhao X., He C. (2014). Structure and properties of chitin whisker reinforced chitosan membranes. Int. J. Biol. Macromol..

[78] Smith D.R., Escobar A.P., Andris M.N., Boardman B.M., Peters G.M. (2021). Understanding the Molecular-Level Interactions of Glucosamine-Glycerol Assemblies: A Model System for Chitosan Plasticization. ACS Omega.

[79] Mutmainna I., Tahir D., Gareso P.L., Ilyas S. (2019). Synthesis composite starch-chitosan as biodegradable plastic for food packaging. J. Phys. Conf. Ser..

[80] Hasan M., Rahmayani R.F.I., Minandar (2018). Bioplastic from Chitosan and Yellow Pumpkin Starch with Castor Oil as Plasticizer. IOP Conf. Ser. Mater. Sci. Eng..

[81] Sapei L., Pdmawijaya K.S., Sijayanti O., Wardhana P.J. (2015). The effect of banana starch concentration on the properties of chitosan-starch bioplastics. J. Chem. Pharm. Res..

[82] Fathanah U., Lubis M.R., Nasution F., Masyawi M.S. (2018). Characterization of bioplastic based from cassava crisp home industrial waste incorporated with chitosan and liquid smoke. IOP Conf. Ser. Mater. Sci. Eng..

[83] Choo K.W., Lin M., Mustapha A. (2021). Chitosan/acetylated starch composite films incorporated with essential oils: Physiochemical and antimicrobial properties. Food Biosci..

[84] Castro J.I., Valencia-Llano C.H., Zapata M.E.V., Restrepo Y.J., Hernandez J.H.M., Navia-Porras D.P., Valencia Y., Valencia C., Grande-Tovar C.D. (2021). Chitosan/Polyvinyl Alcohol/ Tea Tree Essential Oil Composite Films for Biomedical Applications. Polymers.

[85] Jannah M., Ahmad A., Hayatun A., Taba P., Chadijah S. (2019). Effect of filler and plastisizer on the mechanical properties of bioplastic cellulose from rice husk. J. Phys. Conf. Ser..

[86] Babaee M., Garavan F., Rehman A., Jafarazadeh S., Amini E., Cacciotti I. (2022). Biodegradability, physical, mechanical, and antimicrobial attributes of starch nanocomposites containing chitosan nanoparticles. Int. J. Biol. Macromol..

[87] Pratama J.P., Amalia A., Rohmah R.L., Saraswati T.E. (2020). The extraction of cellulose powder of water hyacinth (*Eichhornia crassipes*) as reinforcing agents in bioplastic. IOP. Conf. Ser..

[88] Blilid S., Kedzierska M., Kilowska K., Wronska N., El Achaby M., Katir N., Belamie E., Alonso B., Lisowska K., Lahcini M. (2020). Phosphorylated Micro- and Nanocellulose-Filled Chitosan Nanocomposites as Fully Sustainable, Biologically Active Bioplastics. ACS Sustain. Chem. Eng..

[89] Kongkaoroptham P., Piroonpan T., Pasanphan W. (2021). Chitosan nanoparticles based on their derivatives as antioxidant and antibacterial additives for active bioplastic packaging. Carbohydr. Polym..

[90] Hammi N., Wroska N., Katir N., Lisowska K., Marcotte N., Cacciaguerra T., Bryszewska M., El Kadib A. (2018). Supramolecular Chemistry Driven Preparation of Nanostructured, Transformable and Biologically-active Chitosan-Clustered Single, Binary and Ternary Metal Oxide Bioplastics. ACS Appl. Bio Mater..

[91] Sapei L., Padmawijaya K.S., Sujayanti O., Wardhana P.J. (2017). Study of the influence of ZnO addition on the properties of chitosan-banana starch bioplastics. IOP Conf. Ser. Mater. Sci. Eng..

[92] Nguyen T.H., Boonyaritthongchai P., Buanong M., Supapvanich S., Wongs-Aree C. (2021). Edible coating of chitosan ionically combined with κ-carrageenan maintains the bract and postharvest attributes of dragon fruit (*Hylocereus undatus*). Int. Food Res. J..

[93] Song D.-H., Hoa V.B., Kim H.W., Khang S.M., Cho S.-H., Ham J.-S., Seol K.-H. (2021). Edible Films on Meat and Meat Products. Coatings.

[94] Candra A., Kaban J., Ginting M. (2021). Antimicrobial activity and physical properties of chitosan-aren seed (*Arenga pinnata*) starch edible film. Rasayan J. Chem..

[95] Fernandez J.G., Ingber D.E. (2014). Manufacturing of Large-Scale Functional Objects Using Biodegradable Chitosan Bioplastic. Macromol. Mater. Eng..

[96] Heidari M., Khomeiri M., Yousefi H., Rafieian M., Kashiri M. (2020). Chitin nanofiber-based nanocomposites containing biodegradable polymers for food packaging applications. J. Consum. Prot. Food Saf..

[97] Salaberria A.M., Diaz R.H., Labidi J., Fernandes S.C.M. (2015). Role of chitin nanocrystals and nanofibers on physical, mechanical and functional properties in thermoplastic starch films. Food Hydrocoll..

[98] Robles E., Salaberria A.M., Herrera R., Fernandes S.C.M., Labidi J. (2016). Self-bonded composite films based on cellulose nanofibers and chitin nanocrystals as antifungal materials. Carbohydr. Polym..

[99] Qin Y., Zhang S., Yu J., Yang J., Xiong L., Sun Q. (2016). Effects of chitin nano-whiskers on the antibacterial and physiochemical properties of maize starch films. Carbohydr. Polym..

[100] Salaberria A.M., Diaz R.H., Labidi J., Fernandes S.C.M. (2015). Preparing valuable renewable nanocomposite films based exclusively on oceanic biomass–chitin nanofillers and chitosan. React. Funct. Polym..

[101] Jayakumar R., Prabaharan M., Kumar P.T.S., Nair S.V., Tamura H. (2011). Biomaterials based on chitin and chitosan in wound dressing applications. Biotechnol. Adv..

[102] Satitsri S., Muanprasat C. (2020). Chitin and Chitosan Derivatives as Biomaterial Resources for Biological and Biomedical Applications. Molecules.

[103] Adres Y., Giraud L., Gerente C., Le Cloirec P. (2007). Antibacterial Effects of Chitosan Powder: Mechanisms of Action. Environ. Technol..

[104] He H., Sun C., Weng Y., Huang H., Ni P., Fang Y., Xu R., Wang Z., Liu H. (2022). Catechol modification of non-woven chitosan gauze for enhanced hemostatic efficacy. Carbohydr. Polym..

[105] Pang J., Bi S., Kong T., Luo X., Zhou Z., Qiu K., Huang L., Chen X., Kong M. (2020). Mechanically and functionally strengthened tissue adhesive of chitin whisker complexed chitosan/dextran derivatives based hydrogel. Carbohydr. Polym..

[106] Fakhri E., Eslami H., Maroufi P., Pakdel F., Taghizadeh S., Ganbarov K., Yousefi M., Tanomand A., Yousefi B., Mahmoudi S. (2020). Chitosan biomaterials application in dentistry. Int. J. Biol. Macromol..

[107] Madhumathi K., Kumar P.T.S., Kavya K.C., Furuike T., Tamura H., Nair S.V., Jayakumar R. (2009). Novel chitin/nanosilica composite scaffolds for bone tissue engineering applications. Int. J. Biol. Macromol..

[108] Christy P.N., Basha S.K., Kumari V.S. (2022). Nano zinc oxide and nano bioactive glass reinforced chitosan/poly(vinyl alcohol) scaffolds for bone tissue engineering application. Mater. Today Commun..

[109] Karimipour-Fard P., Jeffrey M.P., Taggert H.J., Pop-Illiev R., Rizvi G. (2021). Development, processing and characterization of Polycaprolactone/Nano-Hydroxyapatite/Chitin-Nano-Whisker nanocomposite filaments for additive manufacturing of bone tissue scaffolds. J. Mech. Behav. Biomed. Mater..

[110] Chang H.K., Yang D.H., Ha M.Y., Kim H.J., Kim C.H., Kim S.H., Choi J.W., Chun H.J. (2022). 3D printing of cell-laden visible light curable glycol chitosan bioink for bone tissue engineering. Carbohydr. Polym..

[111] Liao J., Hou B., Huang H. (2022). Preparation, properties and drug controlled release of chitin-based hydrogels: An updated review. Carbohydr. Polym..

[112] Hoang H.T., Vu T.T., Karthika V., Jo S.H., Jo Y.J., Seom J.W., Oh C.W., Park S.H., Lim K.T. (2022). Dual cross-linked chitosan/alginate hydrogels prepared by Nb-Tz ‘click’ reaction for pH responsive drug delivery. Carbohydr. Polym..

[113] Xia D., Wang F., Pan S., Yuan S., Liu Y., Xu Y. (2021). Redox/pH-Responsive Biodegradable Thiol-Hyaluronic Acid/Chitosan Charge-Reversal Nanocarriers for Triggered Drug Release. Polymers.

[114] Schmuhl R., Krieg H.M., Keizer K. (2001). Adsorption of Cu (II) and Cr (VI) ions by chitosan: Kinetics and equilibrium studies. Water SA.

[115] Boulaiche W., Hamdi B., Trari M. (2019). Removal of heavy metals by chitin: Equilibrium, kinetic and thermodynamic studies. Appl. Water Sci..

[116] Siahkamari M., Jamali A., Sabzevari A., Shakeri A. (2016). Removal of Lead (II) ions from aqueous solutions using biocompatible polymeric nano-adsorbents: A comparative study. Carbohydr. Polym..

[117] Wu J., Cheng X., Lu Y., Yang G. (2019). Constructing biodegradable nanochitin-contained chitosan hydrogel beads for fast and efficient removal of Cu(II) from aqueous solution. Carbohydr. Polym..

[118] Yan L., Li P., Zhou W., Wang Z., Fan X., Chen M., Fang Y., Liu H. (2019). Shrimp Shell-Inspired Antifouling Chitin Nanofibrous Membrane for Efficient Oil/Water Emulsion Separation with In Situ Removal of heavy Metal Ions. ACS Sustain. Chem. Eng..

[119] Zhang X., Elsayed I., Navaranthna C., Schueneman G.T., Hassan E.B. (2019). Biohybrid Hydrogel and Aerogel from Self-Assembled Nanocellulose and Nanochitin as a High-Efficiency Adsorbent for Water Purificaiton. ACS Appl. Mater. Interfaces.

[120] Doan C.T., Tran T.N., Wang C.L., Wang S.L. (2020). Microbial Conversion of Shrimp Heads to Proteases and Chitin as an Effective Dye Adsorbent. Polymers.

[121] Xu J., Zhang Y., He J., Wu J., Li W., Zhang H., Wang H., Tu J., Zhou Y., Dong Y. (2021). Natural and Sustainable Superhydrophobic Nanochitin Aerogels for Collecting Methane Bubbles from Underwater. ACS Sustain. Chem. Eng..

[122] Song Z., Li G., Guan F., Liu W. (2018). Application of Chitin/Chitosan and Their Derivatives in the Papermaking Industry. Polymers.

[123] Zhang T., Kuga S., Wu M., Huang Y. (2020). Chitin Nanofibril-Based Flame Retardant for Paper Application. ACS Sustain. Chem. Eng..

[124] Kadokawa J., Idenoue S., Yamamoto K. (2020). Fabricating Chitin Paper from Self-Assembled Nanochitins. ACS Sustain. Chem. Eng..

[125] Naghdi T., Golmohammadi H., Yousefi H., Hosseinfard M., Kostiv U., Horák D., Merkoçi A. (2020). Chitin Nanofiber Paper Towards Optical (bio)sensing Applications. ACS Appl. Mater. Interfaces.

[126] Aranaz I., Acosta N., Civera C., Elorza B., Mingo J., Castro C., de los Llanos Gandía M., Caballero A.H. (2018). Cosmetics and Cosmeceutical Applications of Chitin, Chitosan and Their Derivatives. Polymers.

[127] Theerawattanawit C., Phaiyarin P., Wanichwecharungruang S., Noppakun N., Asawanonda P., Kumtornrut C. (2021). The Efficacy and Safety of Chitosan on Facial Skin Sebum. Ski. Pharmacol. Physiol..

[128] Azimi B., Ricci C., Fusco A., Zavagna L., Linari S., Donnarumma G., Hadrich A., Cinelli P., Coltelli M.-B., Danti S. (2021). Electrosprayed Shrimp and Mushroom Nanochitins on Cellulose Tissue for Skin Contact Application. Molecules.

[129] Su Z., Yang Y., Huang Q., Chen R., Ge W., Fang Z., Huang F., Wang X. (2022). Designed biomass materials for “green” electronics: A review of materials, fabrications, devices, and perspectives. Prog. Mater. Sci..

[130] Dong Z., Chen C., Wen K., Zhao X., Guo X., Zhou Z., Chang G., Zhang Y., Dong Y. (2022). A Freestanding Chitin-Derived Hierarchical Nanocomposite for Developing Electrodes in Future Supercapacitor Industry. Polymers.

[131] Wang S., Yu W., Chen Y., He J., Zhao Z., Lu Y., Yang Q., Xiong C., Shi Z. (2022). Electrode materials from cuprous oxide and chitin nanofibrils for supercapacitors with high specific capacity. Ionics.

[132] Wang Y., Chen G., Yang F., Luo Z., Yuan B., Chen X., Wang L. (2022). Serendipity discovery of fire early warning function of chitosan films. Carbohydr. Polym..

[133] Yang F., Yuan B., Wang Y., Chen X., Wang L., Zhang H. (2021). Graphene oxide/ chitosan nano-coating with ultrafast fire-alarm response and flame-retardant property. Polym. Adv. Technol..

[134] Shamshina J.L., Kelly A., Oldham T., Rogers R.D. (2019). Agricultural uses of chitin polymers. Environ. Chem. Lett..

[135] Zhou Y., Jiang S., Jiao Y., Wang H. (2018). Synergistic Effects of Nanochitin on Inhibition of Tobacoo Root Rot Disease. Int. J. Biol. Macromol..

[136] Zhou Y., Jing M., Levy A., Wang H., Jiang S., Dou D. (2020). Molecular mechanism of nanochitin whisker elicits plant resistance against Phytophthora and the receptors in plants. Int. J. Biol. Macromol..

[137] Liang R., Li X., Yuan W., Jin S., Hou S., Wang M., Wang H. (2018). Antifungal activity of nanochitin whisker against crown rot diseases of wheat. J. Agric. Food Chem..

[138] Xue W., Han Y., Tan J., Wang Y., Wang G., Wang H. (2017). Effects of Nanochitin on Enhancement of Grain Yield and Quality of Winter Wheat. J. Agric. Food Chem..

[139] Chenh Y., Wang Y., Han Y., Li D., Zhang Z., Zhu X., Tan J., Wang H. (2019). The Stimulatory Effects of Nanochitin Whiskers on Carbon and Nitrogen Metabolism and on the Enhancement of Grain Yield and Crude Protein of Winter Wheat. Molecules.

[140] Li Z., Wang H., An S., Yin X. (2021). Nanochitin whisker enhances insecticidal activity of chemical pesticide for pest insect control and toxicity. J. Nanobiotechnol..

[141] Li Z., Su L., Wang H., An S., Yin X. (2020). Physiochemical and biological properties of nanochitin-abamectin conjugate for Nactuidae insect pest control. J. Nanopart. Res..

[142] Tzoumaki M.V., Moschakis T., Kiosseoglou V., Biliaderis C.G. (2011). Oil-in-water emulsions stabilized by chitin nanocrystal particles. Food Hydrocoll..

[143] Huan S., Zhu Y., Xu W., McClements D.j., Bai L., Rojas O.J. (2021). Pickering Emulsions via Interfacial Nanoparticle Complexation of Oppositely Charged Nanopolysaccharides. Appl. Mater. Interfaces.

[144] Lv S., Zhou H., Bai L., Rojas O.J., McClements D.J. (2021). Development of food-grade Pickering emulsions stabilized by a mixture of cellulose nanofibrils and nanochitin. Food Hydrocoll..

[145] Yu S., Duan M., Sun J., Jiang H., Zhao J., Tong C., Pang J., Wu C. (2022). Immobilization of phlorotannins on nanochitin: A novel biopreservatie for refregerated sea vass (*Lateolabrax japonicus*) fillets. Int. J. Biol. Macromol..

[146] Kishimoto M., Izawa H., Saimoto H., Ifuku S. (2021). Dyeing of chitin nanofibers with reactive dyes and preparation of their sheets and nanofiber/resin composites. Cellulose.

[147] Lizundia E., Nguyen T.D., Winnick R.J., MacLachlan M.J. (2021). Biomimetic photonic materials derived from chitin and chitosan. J. Mater. Chem. C.

[148] Xu J., Liu L., Yu J., Zou Y., Pei W., Zhang L., Ye W., Bai L., Wang Z., Fan Y. (2022). Simple synthesis of self-assembled nacre-like materials with 3D periodic layers from nanochitin via hydrogelation and mineralization. RSC Green Chem..

